# Ternary ionic liquid–water pretreatment systems of an agave bagasse and municipal solid waste blend

**DOI:** 10.1186/s13068-017-0758-4

**Published:** 2017-03-21

**Authors:** Jose A. Perez-Pimienta, Noppadon Sathitsuksanoh, Vicki S. Thompson, Kim Tran, Teresa Ponce-Noyola, Vitalie Stavila, Seema Singh, Blake A. Simmons

**Affiliations:** 1 Department of Chemical Engineering, Universidad Autónoma de Nayarit, Tepic, Mexico; 2 Department of Chemical Engineering and Conn Center for Renewable Energy Research, University of Louisville, Louisville, KY USA; 3 Biological Systems and Engineering Division, Lawrence Berkeley National Laboratory, Joint BioEnergy Institute, 5885 Hollis Street, Emeryville, CA 94608 USA; 4 Biological and Chemical Processing Department, Idaho National Laboratory, Idaho Falls, ID USA; 5 Department of Biotechnology and Bioengineering, CINVESTAV-IPN, Ciudad de México, Mexico; 6 Biological and Engineering Sciences Center, Sandia National Laboratories, Livermore, CA USA; 7 Energy Nanomaterials Department, Sandia National Laboratories, Livermore, CA USA

**Keywords:** Agave bagasse, Biomass blend, Municipal solid waste, Ionic liquid, Ternary system, IL recycling, Biomass pretreatment

## Abstract

**Background:**

Pretreatment is necessary to reduce biomass recalcitrance and enhance the efficiency of enzymatic saccharification for biofuel production. Ionic liquid (IL) pretreatment has gained a significant interest as a pretreatment process that can reduce cellulose crystallinity and remove lignin, key factors that govern enzyme accessibility. There are several challenges that need to be addressed for IL pretreatment to become viable for commercialization, including IL cost and recyclability. In addition, it is unclear whether ILs can maintain process performance when utilizing low-cost, low-quality biomass feedstocks such as the paper fraction of municipal solid waste (MSW), which are readily available in high quantities. One approach to potentially reduce IL cost is to use a blend of ILs at different concentrations in aqueous mixtures. Herein, we describe 14 IL-water systems with mixtures of 1-ethyl-3-ethylimidazolium acetate ([C_2_C_1_Im][OAc]), 1-butyl-3-ethylimidazolium acetate ([C_4_C_1_Im][OAc]), and water that were used to pretreat MSW blended with agave bagasse (AGB). The detailed analysis of IL recycling in terms of sugar yields of pretreated biomass and IL stability was examined.

**Results:**

Both biomass types (AGB and MSW) were efficiently disrupted by IL pretreatment. The pretreatment efficiency of [C_2_C_1_Im][OAc] and [C_4_C_1_Im][OAc] decreased when mixed with water above 40%. The AGB/MSW (1:1) blend demonstrated a glucan conversion of 94.1 and 83.0% using IL systems with ~10 and ~40% water content, respectively. Chemical structures of fresh ILs and recycle ILs presented strong similarities observed by FTIR and ^1^H-NMR spectroscopy. The glucan and xylan hydrolysis yields obtained from recycled IL exhibited a slight decrease in pretreatment efficiency (less than 10% in terms of hydrolysis yields compared to that of fresh IL), and a decrease in cellulose crystallinity was observed.

**Conclusions:**

Our results demonstrated that mixing ILs such as [C_2_C_1_Im][OAc] and [C_4_C_1_Im][OAc] and blending the paper fraction of MSW with agricultural residues, such as AGB, may contribute to lower the production costs while maintaining high sugar yields. Recycled IL-water mixtures provided comparable results to that of fresh ILs. Both of these results offer the potential of reducing the production costs of sugars and biofuels at biorefineries as compared to more conventional IL conversion technologies.

**Graphical abstract:**
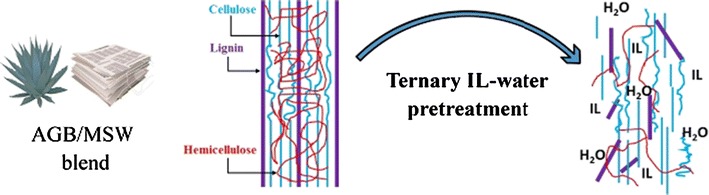
Schematic of ionic liquid (IL) pretreatment of agave bagasse (AB) and paper-rich fraction of municipal solid waste (MSW)

**Electronic supplementary material:**

The online version of this article (doi:10.1186/s13068-017-0758-4) contains supplementary material, which is available to authorized users.

## Background

Liquid transportation fuels and value-added products can be obtained from renewable sources such as grasses and agricultural or forestry residues due to their naturally high carbohydrate content. Moreover, these lignocellulosic biomass materials are available at significant levels and can achieve high sugar production with minimal impact on food sources when compared to first-generation technologies [1]. A pretreatment step is a necessary prerequisite to increase biomass digestibility by reducing its recalcitrance. After this stage, pretreated materials are enzymatically digested into fermentable sugars that are then suitable for biofuel and/or renewable chemical production using fermentation [2]. Various biomass pretreatment technologies have been developed with the general objective to alter or remove hemicellulose and/or lignin, increase surface area and/or decrease the crystallinity of cellulose [3].

In recent years, numerous studies have shown that imidazolium-based ionic liquids (ILs) are attractive as green solvents for biomass pretreatment due to several traits, including high cellulose solubility, low vapor pressure, chemical and thermal stability, non-flammability, and phase behavior. These ILs are relatively benign to the environment when compared to pretreatments that use acids, bases, and/or organic solvents. After IL pretreatment, cellulose can be easily recovered by the addition of an antisolvent, such as water or ethanol [4, 5]. In addition, ionic liquids have been used in the dissolution and partial delignification of corn stover, switchgrass, agave bagasse, softwood, hardwood, and municipal solid waste (MSW) [6–11].

Although IL pretreatment leads to enhanced biomass saccharification, the biggest challenge for commercialization of this technology lies in the relatively high cost of ILs, which can range from $1 up to $800/kg, depending on the purity and source, making it essential to develop comprehensive strategies for improving the overall economics of the biorefineries using IL pretreatment platform [12]. To address the high-cost issue of ILs, we have taken four different approaches into consideration.

The first approach entails the use of MSW (which paper mix represents 30% of total) as a lower quality feedstock; therefore by blending a paper-rich fraction of MSW with a higher quality feedstock overall costs can be reduced in a biorefinery scheme [9, 13]. Currently, most biomass conversion studies have focused on the conversion of a single feedstock with little consideration on feedstock diversity and mixed feedstocks. Moreover, biomass availability varies significantly from region to region due to weather conditions and crop varieties and increase the need for a biorefinery that can effectively and efficiently process mixed feedstocks [14, 15].

A second approach for improving the economics of IL pretreatment involves the utilization of aqueous solutions of ILs as opposed to a typical process that uses 100% IL. These aqueous mixtures have significantly lowered viscosities relative to neat ILs, making handling easier and enhancing mass transfer. Previous findings have shown that selected ILs can act effectively in the presence of water, enhancing glucan digestibility due to competitive hydrogen bonding [16–20]. Decreasing IL use without decreasing sugar yields will be reflected in final production costs.

A third approach used to minimize associated costs with using ILs to pretreat biomass is to employ ILs combination of acetate (anion) and imidazolium (cation) such as [C_2_C_1_Im][OAc] and [C_4_C_1_Im][OAc] which demonstrate high lignin removal and cellulose decrystallization in studies where their specific interactions and performance were examined [7, 11, 21, 22].

Imidazolium-based ILs typically have numerous advantages in biomass biorefineries including pretreatment performance independent of biomass type, moderate reaction values (time and temperature), and compatibility with pretreatment reactor construction materials [23]. Currently, [C_4_C_1_Im][OAc] costs about 80% when compared to [C_2_C_1_Im][OAc], which can lead to reduced costs if pretreatment performance can be maintained.

Finally, a fourth approach concerns the recyclability and reusability of ILs for several consecutive batches, which will likely be required for commercial use as a biomass pretreatment within a biorefinery. Recycling of ILs would occur after the addition of an antisolvent such as water that precipitates cellulose and allows easy recovery through filtration or centrifugation. A number of reports have studied the recovery and recycling of ILs after biomass pretreatment with different conditions and equipment [24–26], addition of kosmotropic anions (such as phosphate carbonate and sulfate) to form aqueous biphasic systems [27, 28] and from a technoeconomic perspective [29]. Nevertheless, the particular effects on the recycled ILs and its impact on biomass pretreatment have not been completely elucidated.

**Fig. 1:**
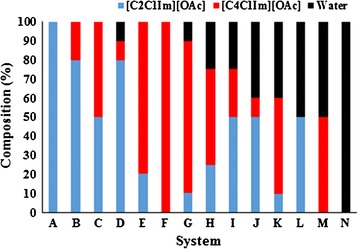
Aqueous ionic liquid systems employed in the pretreatment of agave bagasse (AGB), municipal solid waste (MSW), and an AGB/MSW (1:1) blend

This study aims to assess the effect of a ternary aqueous system by mixing [C_2_C_1_Im][OAc], [C_4_C_1_Im][OAc], and water at 14 selected ratios for the pretreatment of a 1:1 blend of MSW and agave bagasse (AGB) (Fig. 1). AGB was selected to be blended with MSW due to favorable characteristics as a bioenergy feedstock such as high carbohydrate content, low water inputs, and high productivities in semiarid regions as well as previous studies that demonstrated high sugar yields can be obtained after IL pretreatment [8]. In order to better understand the pretreatment process, changes in chemical structure were examined by Fourier Transform Infrared (FTIR) spectroscopy, ^1^H NMR, and component characterization. We also examined the effects of recycling [C_2_C_1_Im][OAc] and [C_4_C_1_Im][OAc] three times on pretreatment performance. To the best of our knowledge, this is the first report that employs a mixture of ILs and water for the pretreatment of mixed feedstock blends.

## Methods

### Materials and preparation

For the MSW, paper waste materials were prepared as in [9], consisting of 15% glossy paper, 25% non-glossy paper, 32% non-glossy cardboard, and 28% glossy cardboard using a process developed by Idaho National Laboratory (INL). It is recognized that this material is not representative of real MSW streams and that there may be contaminants present that will impact pretreatment effectiveness. However, the goal of this study was to examine the effectiveness of the IL systems in this study on the types of paper that would be found in MSW. Destiladora Rubio, a tequila plant from Jalisco, Mexico, donated the AGB. The AGB was milled with a Thomas-Wiley Mini Mill fitted with a 40-mesh screen (Model 3383-L10 Arthur H. Thomas Co., Philadelphia, PA, USA). Both ground biomass samples were stored at 4 °C in a sealed plastic bag prior to their use. The 1:1 blend was prepared by mixing both MSW and AGB in the pretreatment reactor just before the heating process begins. 1-ethyl-3-methylimidazolium acetate [C_2_C_1_Im][OAc] and 1-butyl-3-methylimidazolium acetate [C_4_C_1_Im][OAc], citric acid, ethanol, glucose, xylose, sulfuric acid, and HPLC grade water were purchased from Sigma–Aldrich.

### Aqueous ionic liquid pretreatment in tube reactors

A design of experiments was carried out using Minitab^®^ software (Coventry, UK), utilizing 14 unique aqueous ionic liquid combinations (ranked in cost decreasing order), composed of two ionic liquid: [C_2_C_1_Im][OAc] and [C_4_C_1_Im][OAc] plus DI water (constrained up to 50% when combined with ILs) at different ratios (Fig. 1). One gram of biomass (dry basis) was mixed with 9 g of the specific ternary aqueous IL solution to give a 10% (w/w) biomass solution. The biomass was loaded in tubular reactors made of 0.75-in diameter × 6-in length Hastelloy (C276) tubes, which were then sealed with stainless steel caps. All pretreatment procedures were run in triplicate in tubular reactors that were heated to reaction temperature (120 °C) for 3 h in a WVR convection oven [8]. After pretreatment, all reactors were quenched by quickly transferring them to a room temperature water bath until the temperature dropped to 30 °C, followed by a washing step performed as previously described [30]. A total of 42 experiments were carried out, where the recovered product was lyophilized for two days in a FreeZone12 (Labconco, MO, USA) equipment before compositional analysis.

### Recycle of ionic liquid and pretreatment

The IL/water mixtures obtained from the pretreatments in tube reactors with pure [C_2_C_1_Im][OAc] and [C_4_C_1_Im][OAc] in AGB were evaporated at 100 °C for 12 h in a drying oven to remove excess water, and then reused to pretreat AGB in tube reactors at 120 °C and 3 h at ambient pressure without any further purification. A total of 3 cycles were performed where the solution of each IL was again separated, concentrated, and reused. The recycling pretreatments were conducted in duplicate and the IL/biomass mixture was homogenized using a glass rod. A portion of the recovered biomass on each cycle was stored for compositional analysis and other one was used for enzymatic saccharification. For each IL recycle, 500 µL was withdrawn to analyze their integrity by FTIR and ^1^H-NMR.

### Chemical characterization

Sugars content of untreated and pretreated biomass samples were determined according to the standard analytical procedures of the National Renewable Energy Laboratory (NREL) LAP 017 using a two-step acid hydrolysis method [31]. Briefly, for all samples, 0.3 g of dry biomass was treated with 3 mL of 72% H_2_SO_4_ for 60 min at 30 °C with constant agitation, then diluted with 84 mL of DI water, finally autoclaved at 121 °C for 1 h. The content of acid insoluble lignin (referred to as lignin in the rest of the manuscript) was determined gravimetrically as the solid residue remaining after two-step hydrolysis. The liquid filtrates were used to determine the carbohydrate concentrations by Agilent HPLC 1200 series equipped with a Bio-Rad Aminex HPX-87H column and a refractive index detector.

Delignification was calculated using the following equation:

1 $${\text{Delignification (}}{\% } )\;{ = }\frac{{{\text{Initial lignin}} - {\text{Recovered lignin}}}}{\text{Initial lignin}}\;{ \times }\; 1 0 0.$$

### Enzymatic saccharification

Saccharification of all biomass samples was carried out at 55 °C and 150 rpm for 72 h in 50 mM citrate buffer (pH 4.8) in a rotary incubator with commercial enzyme cocktails, Cellic^®^ CTec2 and HTec2, obtained as a gift from Novozymes. The protein content of enzymes was determined by bicinchoninic acid (BCA) assay with a Pierce BCA Protein Assay Kit (Thermo Scientific) using BSA as protein standard. CTec2 has a protein content of 186.6 ± 2.0 mg/mL, and protein content of HTec2 was 180.1 ± 1.8 mg/mL. The enzyme activity of CTec2 was determined to be ~80 filter paper units (FPU)/mL. The enzyme loading was normalized to the glucan content (5 g/L) present in the biomass samples to understand the impact of each pretreatment in the response variable of sugar production. Hence, the enzyme concentration of CTec2 and HTec2 was set constant at 20 mg protein/g glucan and 2 mg protein/g xylan, respectively. All assays were performed in triplicate.

### Analysis of saccharified samples

Sugars concentrations were monitored using HPLC by taking 50 µL of the saccharification supernatant. The samples were filtered in 0.45 µm Pall 96-well filter plate, centrifuged (4000 rpm—5 min), recollected in a 96-well Bio-Rad plate and finally covered with pierceable aluminum foil (to prevent vapor losses) to monitored glucose and xylose production in all samples by an Agilent HPLC 1200 series equipped with a Bio-Rad Aminex HPX-87H column and a refractive index detector. The glucan conversion was calculated using

2 $$\begin{aligned} {\text{Glucan conversion (}}\% ) & = \frac{{{\text{Glucose conc }}( {{{{\text{g}}} /{{{\text{mL}}}}}}) \times {\text{Reaction vol }}( {{\text{mL}}})}}{{{\text{Biomass }}( {\text{g}}) \times {\text{wt}}\% \,{\text{cellulose in biomass}}}} \\ & \quad \times \frac{{162\;({\text{PM glucan unit)}}}}{{180\;({\text{PM glucose unit)}}}} \times 100\end{aligned}$$

and is based on the mass of each material used before pretreatment, thus representing an overall process conversion. The xylan conversion was calculated using

3 $$\begin{aligned}{\text{Xylan conversion }}(\% ) &= \frac{{{\text{Xylose conc }} ({{{\text{g}}/{{\text{mL}}}}}) \times {\text{Reaction vol }}\left( {{\text{mL}}} \right)}}{{{\text{Biomass }}( {\text{g}}) \times {\text{wt}}\% \;{\text{xylan in biomass}}}} \\ & \quad \times \frac{{132\;({\text{PM xylan unit}})}}{{150\;({\text{PM xylose unit}})}} \times 100 \end{aligned}$$

and is based on the difference in molecular weight between xylan and the xylose unit [32].

### Attenuated total reflectance (ATR)-FTIR spectroscopy

ATR-FTIR was conducted using a Bruker Optics Vertex system with built-in diamond-germanium ATR single reflection crystal. All samples were pressed uniformly against the diamond surface using a spring-loaded anvil. Sample spectra were obtained in triplicates using an average of 128 scans over the range between 800 and 2000 cm^−1^ with a spectral resolution of 4 cm^−1^. Air, water, and the appropriate IL solution were used as background for untreated and pretreated biomass samples, respectively. Baseline correction was conducted using the rubber band method following the spectrum minima [5].

### Crystallinity measurement

XRD diffractogram of untreated and IL-treated AGB with fresh and recycled ILs ([C_2_C_1_Im][OAc] and [C_4_C_1_Im][OAc]) in AGB were acquired with a PANalytical Empyrean diffractometer equipped with a PIXcel3D detector with Cu Kα radiation. The samples were scanned in the range of 5–50° (2θ) with a step size of 0.026° at 45 kV and 40 mA under ambient temperature. Crystallinity index (CrI) was calculated by using Eq. (4) [33]

4 $${\text{CrI}} = \frac{{I_{002} - I_{\text{am}} }}{{I_{002} }},$$

where *I*_002_ is the intensity for the crystalline portion of biomass at about 2θ  =  22.4, and *I*_am_ is the peak for the amorphous portion at 2*θ* =  16.6.

### Proton nuclear magnetic resonance (^1^H-NMR) spectroscopy

^1^H NMR spectra of fresh and recycled ILs were acquired at 25 °C using a Bruker DRX-500 MHz instrument equipped with a Z-gradient inverse TXI ^1^H/^13^C/^15^N 5 mm probe (ns = 128 and d_1_ = 10.0 s). Chemical shifts were referenced to tetramethylsilane. The NMR spectra were processed using Bruker’s Topspin 3.1 (Windows) processing software.

### Statistical analysis

The software Minitab 17 was used for analysis of variance (ANOVA) of experimental results. A 5% probability level (*p* = 0.05) was used to accept or reject the null hypothesis of significant differences. Duncan’s multiple range test at the level of 5% was used to analyze the significances of glucan and xylan conversion of the pretreated biomass besides delignification and glucan conversion of the effect of using recycled ILs [34].

## Results and discussion

### Compositional analysis of untreated and pretreated biomass

The initial step to decrease biomass recalcitrance towards fermentable sugars, this is to pretreat the feedstock for downstream processing (saccharification and fermentation). Previous studies have found that [C_2_C_1_Im][OAc] is an effective solvent to solubilize AGB bagasse plant cell wall, regenerating cellulose while rejecting lignin upon antisolvent addition with optimal conditions for AGB at 120 °C for 3 h [8, 30]. To provide lower cost biorefinery feedstock inputs, MSW have been used as a blending agent in different feedstocks (e.g., corn stover) using IL pretreatment with advantageous features such as year-round availability, reduce landfill disposal and meet biorefinery overall quality specifications [9]. Recently, different studies have been carried out to determine the impact and effectiveness on pretreatment technologies of mixed lignocellulosic biomass as the feedstock costs remain a large contributor to biofuel production costs including that each material responds differently to a specific process (e.g., component removal, sugar yield) [29, 35, 36].

**Fig. 2:**
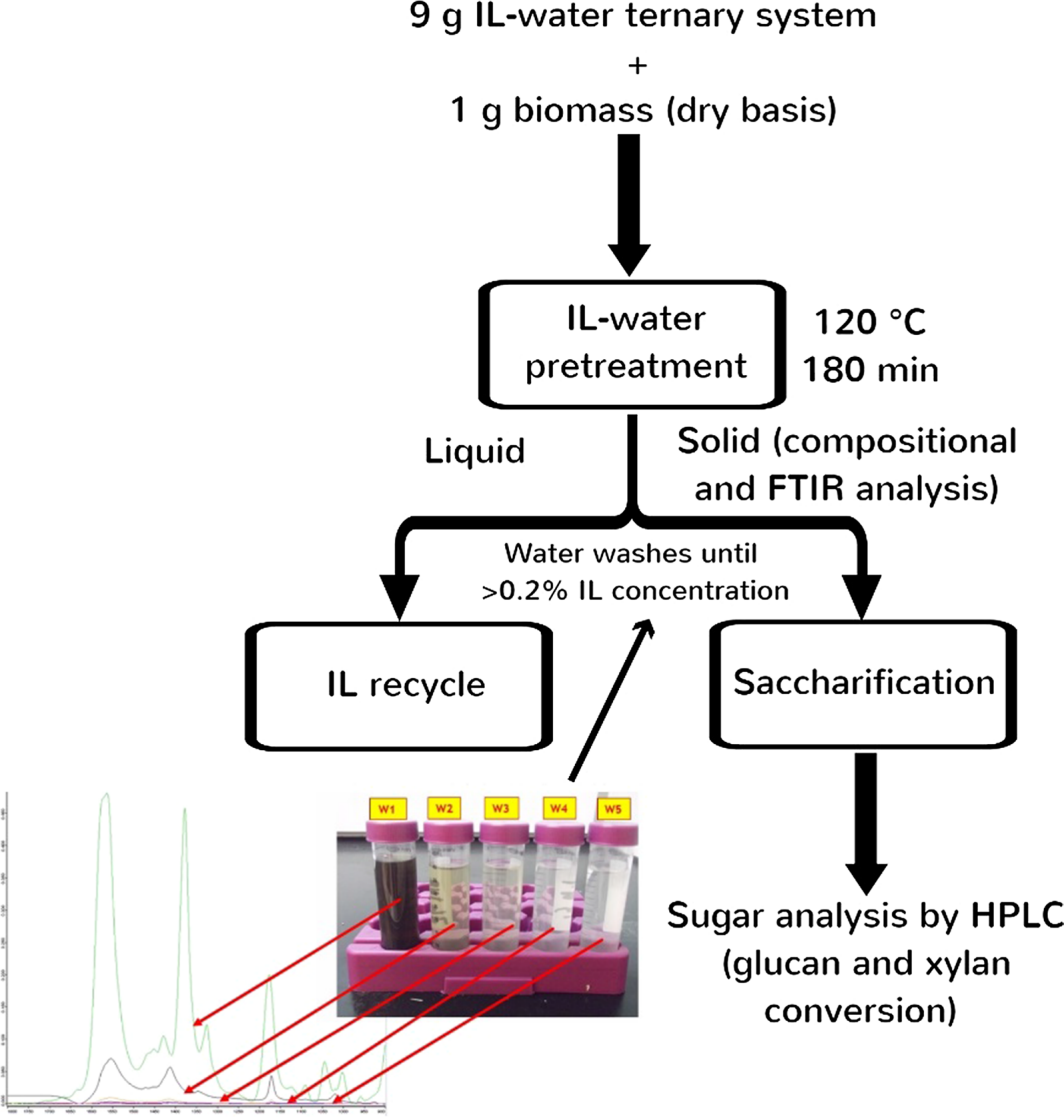
Process flowsheet of the IL-water pretreatment systems on agave bagasse (AGB), municipal solid waste (MSW), and an AGB/MSW (1:1) blend

**Fig. 3:**
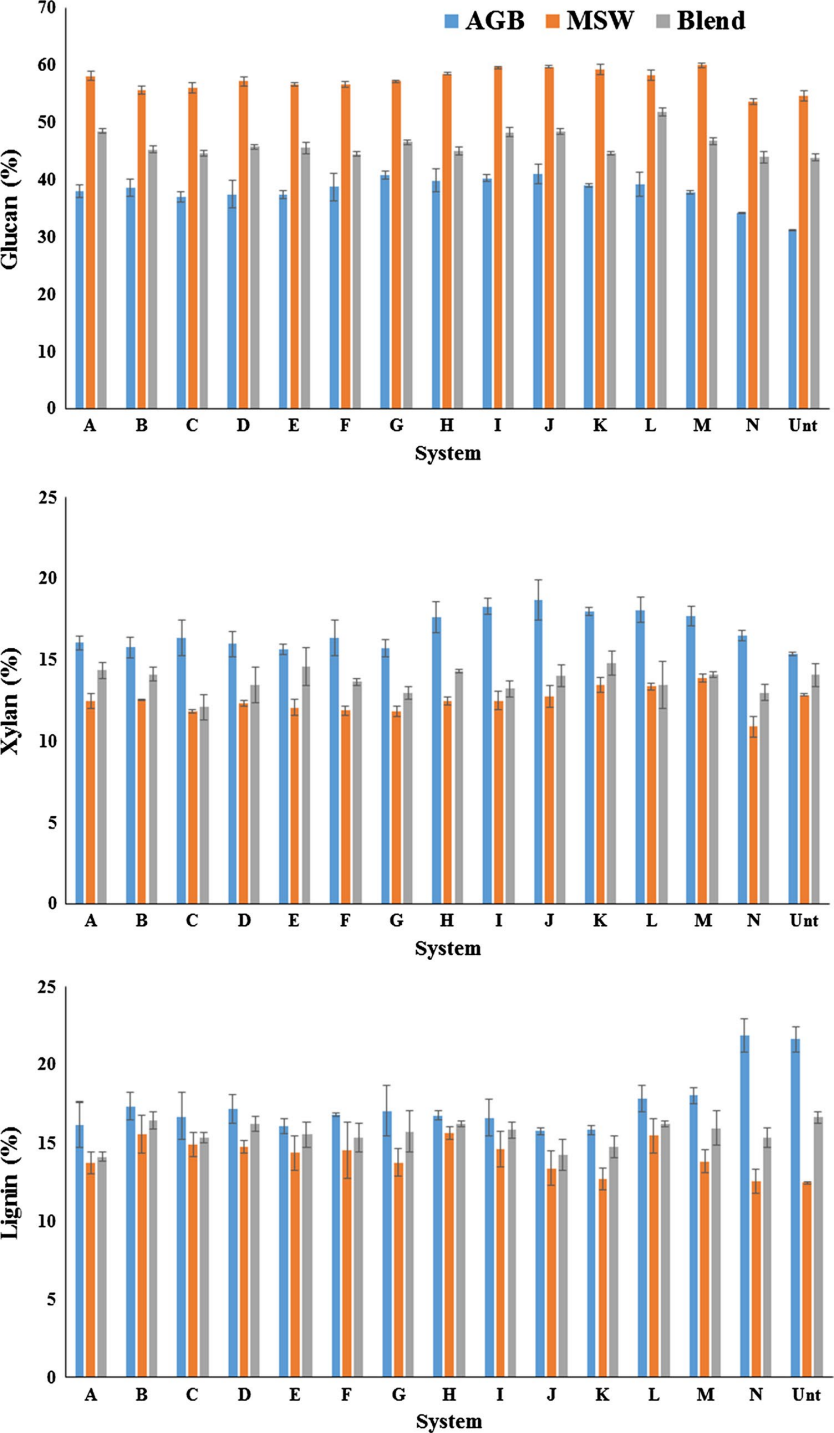
Compositional analysis of untreated and pretreated biomass under different aqueous ionic liquid systems

The process flowsheet of the IL-water pretreatment systems is shown in Fig. 2. Figure 3 presents the compositional analysis of untreated and all 14 IL-water pretreatment system using AGB, MSW, and AGB/MSW (1:1) blend where three major plant cell wall components (glucan, xylan, and lignin) were monitored. For the untreated AGB, a 31.3% glucan, 15.4% xylan, and 21.6% lignin compositional profile measured is comparable to other agave bagasses from the *Tequilana* species, but relatively lower in glucan content and higher in lignin compared to other reported agave compositions which had glucan and lignin values above 40% and under 20%, respectively [37, 38]. This difference can potentially be attributed to process conditions during tequila production and/or environmental conditions of the biomass source, extraction, and post-harvest procedures. Compositional profile of untreated MSW was 54.7% glucan, 12.9% xylan, and 12.5% lignin, similar to that reported by Sun et al. [9] and similar to the individual composition from two constituents of MSW (newspaper and office paper) described by Foyle et al. [39]. As expected, intermediate values were obtained for the AGB/MSW (1:1) blend with 43.9% glucan, 14.1% xylan, and 16.7% lignin.

In order to measure the response of each component from the aqueous IL systems, 100% concentration of [C_2_C_1_Im][OAc], [C_4_C_1_Im][OAc], and water was included in the experimental design as systems A, F, and N, respectively.

Compositional profiles for pretreated samples (Fig. 3) indicate that almost all systems studied achieve a higher glucan content increase (2–24%) with the exception of system N (100% water) where negative values for MSW and AGB/MSW (1:1) blend were obtained. Using system J with ~40% water obtained a ~24% glucan increase with AGB that was higher than the one obtained with system A (18%) when compared to the untreated sample.

A~9% glucan increase using MSW was achieved obtained in three systems (I, J, and M) and was comparable to the trends obtained by Sun et al. [9] that reports an increment of 6% of glucan when compared to the untreated biomass. Finally, the AGB/MSW (1:1) blend increased the glucan content by 15 and ~10% with system L and I, respectively. In terms of xylan content, the pretreated AGB showed a similar trend as in previous reports, increasing its loading from 1 to 18%. As opposite as in MSW, where the general trend shows a xylan reduction up to 9% in the IL-treated samples while the AGB/MSW (1:1) blend presents mix results.

One of the most important features of IL pretreatment is the high levels of delignification that can be achieved. When compared to the untreated AGB, a significant reduction in lignin content was observed after pretreatment. Lignin content was decreased up to 26.9% with system J (~40% water) comparable to that using system A (100% [C_2_C_1_Im][OAc]) which was our base control. Nevertheless, with MSW slight increases were observed with the IL-treated samples and these differences may be attributed to the nature of the lignin in these two feedstocks. A recent study investigated the [C_2_C_1_Im][OAc] dissolution of a corn stover/MSW (1:1) blend at 140 °C from 1 to 3 h, and obtained lignin reduction of 46.2, 69.5, and −0.8% for the blend, corn stover, and MSW, respectively, where a negative number stands for a relative increase on lignin content [9].

Lignin removal from the AGB/MSW (1:1) blend was obtained with system A (15.1%) and system J (14.4%), and were not statistically different. This represents a cost savings since system J uses 40% less IL than system A which is neat IL. In this context, Fu and Mazza [40] presented delignification values of 3.6 and 5.6% with a mixture solution of 1:1 [C_2_C_1_Im][OAc]/water with neat [C_2_C_1_Im][OAc] using Triticale straw. Furthermore, Shi et al. [35] showed that a high sugar yield could be obtained using mixed lignocellulosic feedstocks in which IL pretreatment is capable of handling them with equal efficiency.

Sun et al. [9] attribute the difference on delignification to the nature of lignin in MSW, as this paper mix has already gone through a pulping process that removed most of the lignin although, lignin structure in the MSW is thus expected to be more recalcitrant compared to the intact lignin in AGB making it more difficult to be extracted.

Summarizing, in general terms, system A as expected from neat [C_2_C_1_Im][OAc] presented positive improvement in terms of lignin removal and glucan enrichment from the studied aqueous IL systems and biomass feedstocks while when only water was used (system N), the process temperature (120 °C) was not high enough to substantially modify the biomass cell wall. The intrinsic variation in the cell wall components from the studied materials made that the response on which IL-aqueous system reduce the biomass recalcitrance on higher or lesser magnitude as in the AGB.

### ATR-FTIR analysis

Normalized FTIR spectra between 800 and 2000 cm^−1^ were used to characterize the chemical fingerprints of the feedstocks before and after IL pretreatment (see Additional file 1). For ATR-FTIR data, seven bands are used to monitor the chemical changes of lignin and carbohydrates, and two bands for changes in calcium oxalate intensity in AGB. As expected, the main antisymmetric carbonyl stretching band specific to the oxalate family occurs at 1618 cm^−1^ for calcium oxalate and the secondary carbonyl stretching band, the metal-carboxylate stretch, is located at 1317 cm^−1^. Those two bands are observed to decrease with IL pretreatment in all AGB samples, in agreements with a previous report [30]. Calcium oxalate is located in a large group in the AGB/MSW (1:1) blend but does not appear in MSW. Only AGB presents the 1745 cm^−1^ in a great the intensity and a reduction trend was found in all IL-treated samples. This band is associated with carbonyl C=O stretching, indicating cleavage of lignin and side chains increasing slightly only on system N (100% water). The mixture employed to represent MSW (glossy paper, non-glossy paper, non-glossy cardboard, and glossy cardboard) has an untreated spectrum similar to those obtained from newspaper and paper [41–43]. Typically, the bands at 1510 and 1605 cm^−1^ show the aromatic skeletal vibrations of lignin and are used to reflect the delignification that occurs during IL pretreatment when compared to the untreated spectrum. These bands are assigned for C=O stretching in conjugated p-substituted aryl ketones [44]. In AGB and in some samples of the AGB/MSW (1:1) blend, these bands (1510 and 1605 cm^−1^) are affected by the broad and intense calcium oxalates peaks, which does not occur with MSW. An increase is shown in the IL-treated samples of the band at 1375 cm^−1^ (C–H deformation in cellulose and hemicellulose).

Furthermore, a significant increase of band intensities is observed in all samples at 1056 cm^−1^ (C–O stretch in cellulose and hemicellulose), and the band intensity at 1235 cm^−1^ (C–O stretching in lignin and hemicellulose). In addition to that, the crystalline-to-amorphous cellulose ratio peaks of 1098 and 900 cm^−1^ decreased as a function of IL pretreatment temperature, indicating reduction of cellulose crystallinity in most of pretreated samples when compared to the untreated spectrum [6]. Finally, an increase in the band intensity at 900 cm^−1^ (antisymmetric out-of-plane ring stretch of amorphous cellulose) is observed in the spectra of IL-treated samples, which reflects the relative increase in cellulose content as a result of partial removal of both lignin and hemicellulose in the biomass AGB, MSW, and AGB/MSW (1:1) blend.

### Comparison of the enzymatic saccharification of aqueous IL-treated biomass

**Fig. 4:**
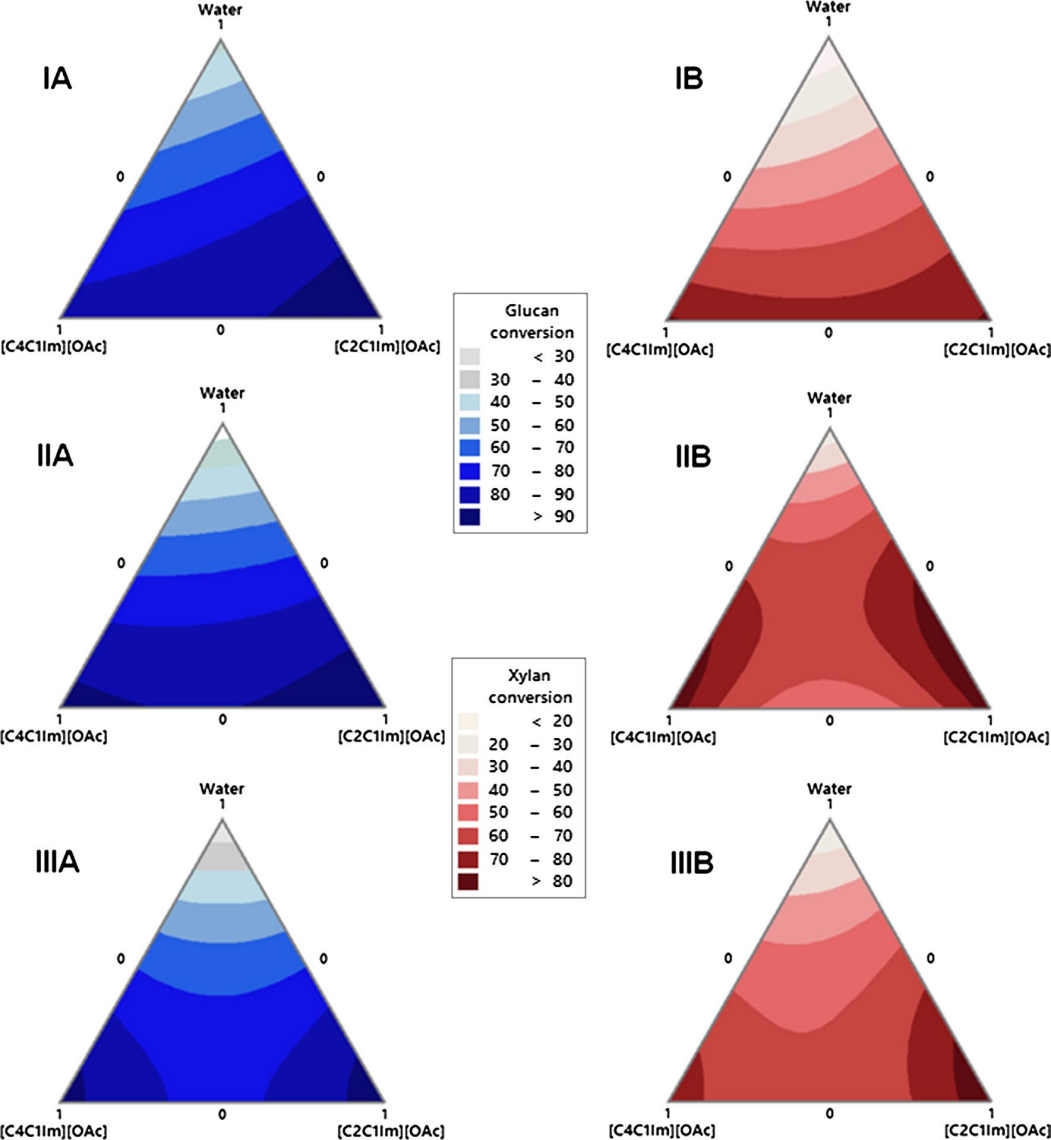
Glucan (*A*) and xylan (*B*) conversion contour plots for ternary ionic liquid systems of [C_2_C_1_Im][OAc], [C_4_C_1_Im][OAc] and water. (*I*) Agave bagasse, (*II*) municipal solid waste, and (*III*) AGB/MSW (1:1) blend

Figure 4 shows the 72 h glucan and xylan conversion of AGB, MSW, and AGB/MSW (1:1) blend. As expected from untreated samples, all three feedstocks showed values under 26 and 14% in terms of xylan conversion. On the other hand, system N (100% water) displayed sugar conversions similar to the untreated samples, as process temperature was not high enough to initiate autohydrolysis.

The AGB using system J had a (97.6%) glucan conversion similar to system A (94.7%) offering an advantage in terms of IL utilization where a relatively high water content (~40%) maintained a sugar conversion comparable to neat IL pretreatment (Fig. 4-IA), correlated with high delignification values. For IL pretreated MSW (Fig. 4-IIA), 96.7% of glucan conversion was obtained with system J, whereas conversion values above 90% were reached when neat systems were employed. When IL-water mixtures were used, System D (10.1% water) achieved a high glucan conversion (~93%), in contrast with system L (50% water) with an 83.1%. Agave bagasse and MSW obtained xylan conversion yields above 87 and 76% for system A and B, respectively (Fig. 4-IB, IIB).

Saccharification of AGB/MSW (1:1) blend showed a 72-h glucan conversion of 96.8% (system A), 94.1% (system D,  ~10% water), and 83.0% (system J, ~40% water) (Fig. 4-IIIA). Hence, a high sugar conversion was obtained using the AGB/MSW (1:1) blend in an IL system with an equal efficiency as that obtained using neat [C_2_C_1_Im][OAc]. In terms of xylan conversion of the AGB/MSW (1:1) blend, 92.2% was obtained using system A, while IL-water systems were in the range of 65–78% (Fig. 4-IIIB). The improved saccharification for IL pretreated samples was due to the decrease biomass recalcitrance granted by weaken the van der Waals interaction between cell wall polymers and disrupt the covalent linkages between hemicellulose and lignin [36].

Table 1 shows a comparison with selected pretreatments that maximize enzymatic digestibility of AGB and MSW. Each pretreatment has it distinctive operation parameters and interaction with the lignocellulosic biomass where IL pretreatment outperformed other processes wits fast saccharification rates and high sugar yields where this difference could be attributed to an improved substrate availability.

**Table 1:** Comparison of selected pretreatments that maximize enzymatic digestibility of agave *tequilana* bagasse (AGB) and municipal solid waste (MSW)—paper mix

Pretreatment	Feedstock	T (°C)	t (min)	Chemical loading (g/g dry biomass)	Removal		Solid loading at saccharification	Sugar conversion at 72 h		Reference
					% Xylan	% Lignin				
								% Glucan	% Xylan	
AFEX	AGB	102	30	2	0	0	5 g glucan/L	81	82	[48]
	MSW	65	15	2.8	0	0	1% glucan	34	47	[49]
Alkaline*	AGB	120^a^ and 24^b^	60^a^ and 1440^b^	0.3^a^ and 0.3^b^	6	83	1:5 solid/liquid	98	48	[50]
Dilute acid	AGB	147	15	0.18	100	−67	8.2 (%, w/w)	68^+^		[51]
	MSW	140	40	0.1	42	79	1% glucan	80	ND	[52]
Hydrothermal	AGB	180	30	–	80	2	5 g glucan/L	80	83	[48]
Ionic liquid [C_2_C_1_Im][OAc]	AGB	120	180	9	7	25	5 g glucan/L	95	92	[48]
	AGB	120	180	9	4	25	5 g glucan/L	95	87	This study—system A
	AGB	120	180	9	18	27	5 g glucan/L	98	80	This study—system J
	MSW	140	180	9	0	6	10% biomass	93	73	[9]
	MSW	120	180	9	0	0	5 g glucan/L	97	94	This study system A
	MSW	120	180	9	0	0	5 g glucan/L	93	75	This study system D
Ionic liquid [C_4_C_1_Im][Cl]	AGB	120	180	9	10	12	5 g glucan/L	82^**+**^		[53]

*AFEX* ammonia fiber expansion, *ND* non determined

* Batch process using consecutive pretreatments with ^a^ NaOH and ^b^ H_2_O_2_

^**+**^Total sugar yield

Overall, all three biomass samples can be efficiently saccharified obtaining a high sugar conversion when compared to the untreated samples, and comparable sugar yields were observed for the IL mixtures relative to those obtained with neat ILs.

A few reports exist where IL-water systems have been used to investigate the dissolution of lignocellulosic biomass using imidazolium-based cation.

Fu and Mazza [40] study the [C_2_C_1_Im][OAc]-water pretreatment of triticale straw at 150 °C for 90 min, and achieved a sugar yield of 81 for 50% water and 67% for neat IL, which were lower than the glucan conversion efficiency of 98% for AGB (40% water) and 83% for MSW (50% water) switchgrass at 120 °C for 3 h in this study. Similarly, Brandt et al. [45] using aqueous solutions applied two ionic liquids (1-butyl-3-methylimidazolium methyl sulfate [C_4_C_1_Im][MeSO_4_] and 1-butyl-3-methylimidazolium hydrogen sulfate [C_4_C_1_Im][HSO_4_]) in *Miscanthus* pulp at 120 °C, and were able to achieve a glucan conversion of 85% and 92% using solutions containing 40% and 10% water content, respectively. Nonetheless, the [C_4_C_1_Im][MeSO_4_] pretreatment was carried out for 22 h, while [C_4_C_1_Im][HSO_4_] pretreatment lasted 13 h, higher processing times values than the 3 h in this study. Another paper reported 88% glucose yield from sugarcane bagasse using [C_4_C_1_Im][Cl] solution containing 20% water and 1.6% H_2_SO_4_ at 130 °C for 30 min [46]. In addition, Shi et al. [47] show that 50–80% [C_2_C_1_Im][OAc]-water mixtures at 160 °C in switchgrass can match the performance of neat [C_2_C_1_Im][OAc] in terms of glucose yield.

Finally, taking into consideration the decreased use of ILs when mixing with up to 40% water, this will impact process economics by reducing associated costs with recycling and handling (with a less viscous solution). This method is also very versatile when employing mixed biomass due to feedstock flexibility, where MSW can provide a lower cost and reduce the environmental impact on subsequent landfill disposal.

### IL recycling

**Fig. 5:**
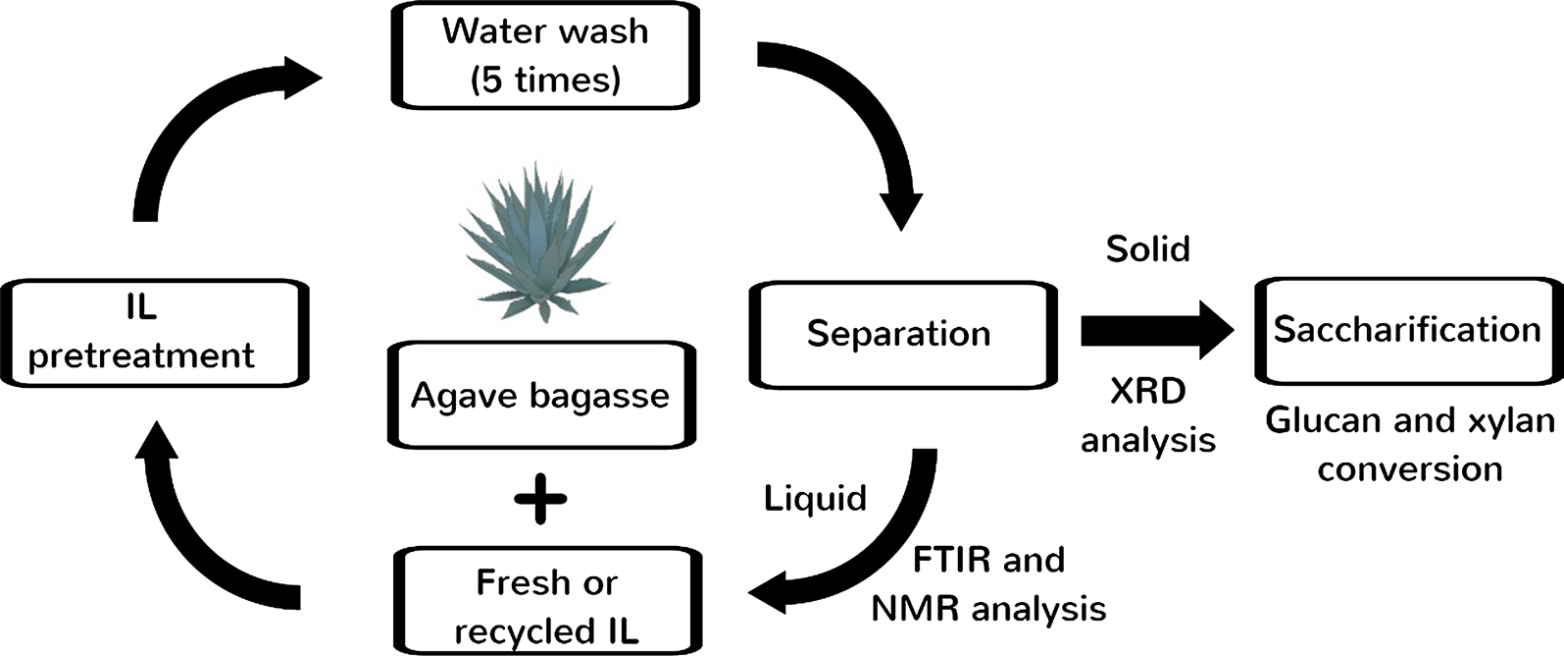
Work flow of ionic liquid recycling of [C_2_C_1_Im][OAc] and [C_4_C_1_Im][OAc] in agave bagasse

**Fig. 6:**
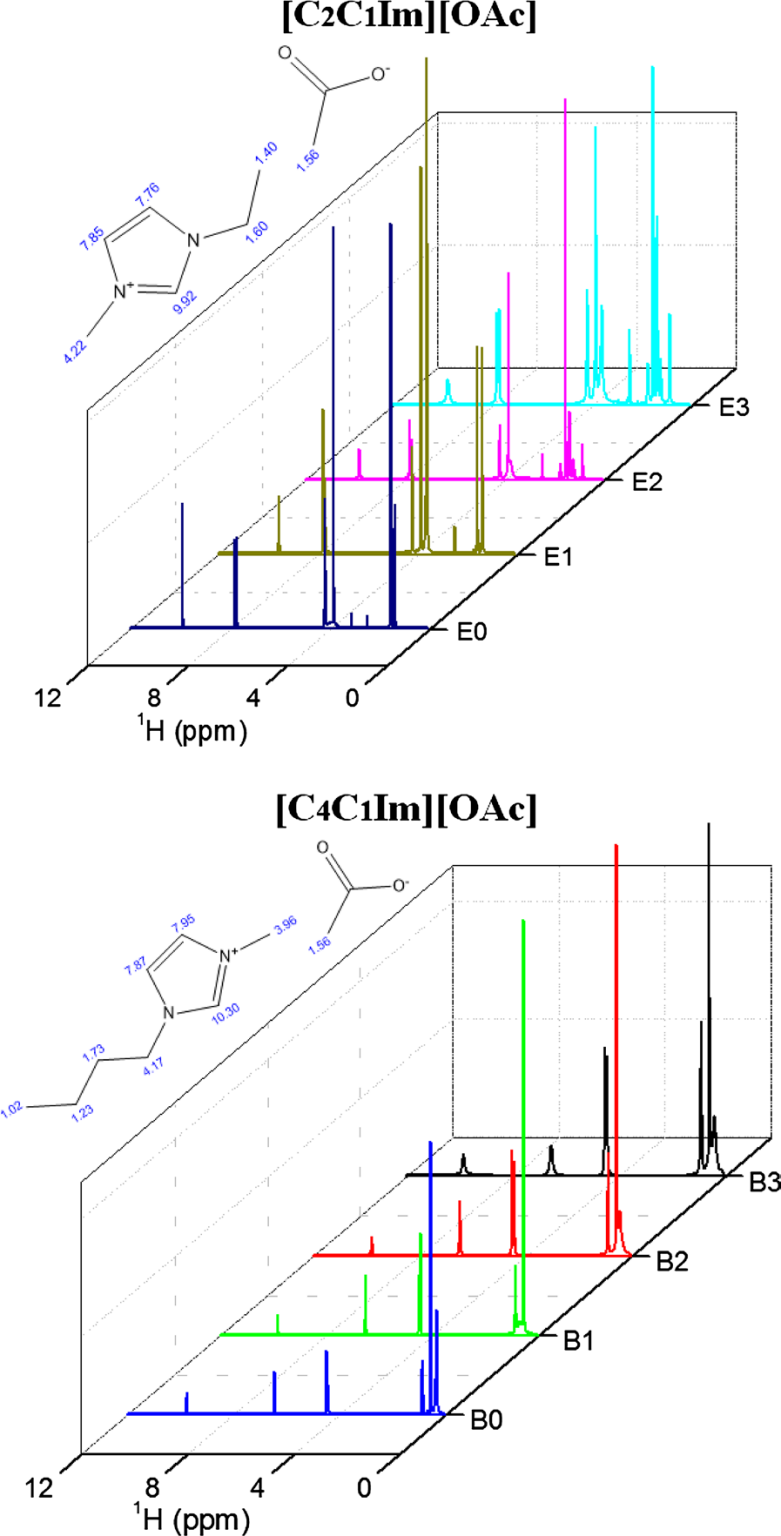
^1^H-NMR analysis of 3 series of recycled [C_2_C_1_Im][OAc] and [C_4_C_1_Im][OAc] in agave bagasse. *0* Fresh ionic liquid, *1* 1st recycle, *2* 2nd recycle, and *3* 3rd recycle

In order to obtain an affordable and scalable IL conversion technology, an efficient process for the recycle and reuse of the ILs is mandatory. In addition, dissolved lignin and/or xylan could be recovered; hence, an added value to the overall process can be attained. The effects of recycled ILs and their impact on biomass pretreatment have not been completely elucidated. By addition of an antisolvent (water), a major fraction of the cellulosic content of the biomass can be recovered from the IL solution forming a single phase. In this study, we used the recovered IL/water mixtures from system A and system B to perform 3 subsequent recycle steps by IL pretreating fresh untreated AGB (120 °C and 3 h), and conclude with a saccharification step (Fig. 5). The IL recycling was performed to test imidazolium-based ionic liquids using only AGB (as a more homogenous sample than MSW), to understand the feasibility of pretreatment and possible changes of its molecular structure. Approximately, 85–90% of IL was recovered on each recycle. Figure 6 presents the ^1^H-NMR and Additional file 2 shows the FTIR analysis of 3 series of recycled [C_2_C_1_Im][OAc] and [C_4_C_1_Im][OAc] in AGB. Based on both spectra, [C_2_C_1_Im][OAc] and [C_4_C_1_Im][OAc] appear to hold their structure as shown on their proton spectra and the distinctive FTIR bands (1175, 1378, and 1574 cm^−1^) from fresh ILs to the recycled ones. Recycled [C_2_C_1_Im][OAc] shows an extra peak at 3.6 ppm suggesting that recycled IL contained residual sugars; however, these sugars did not affect its recycle. This may be probably due to relatively severe recycle conditions employed (100 °C—12 h). Nonetheless, this did not have a significant effect on biomass crystallinity of AGB using fresh ILs or recycled ILs. In addition, we have observed methoxyl peak (~2.5 ppm) in the ^1^H NMR, suggesting that the change in color of IL is partly due to the presence of lignin.

The ratios of crystalline to amorphous cellulose and disordered components found in untreated, fresh ILs, and recycled IL were used to determine the crystallinity index (CrI), as cellulose crystallinity has shown to affect the enzymatic saccharification. Both AGB samples pretreated with fresh ILs present a transition from cellulose I polymorph to cellulose II polymorph as the (002) peak around 22.1° was shifted to a lower angle (20.6°) after IL pretreatment (see Additional file 3). The CrI of the pretreated samples decreased when compared to the untreated sample.

**Fig. 7:**
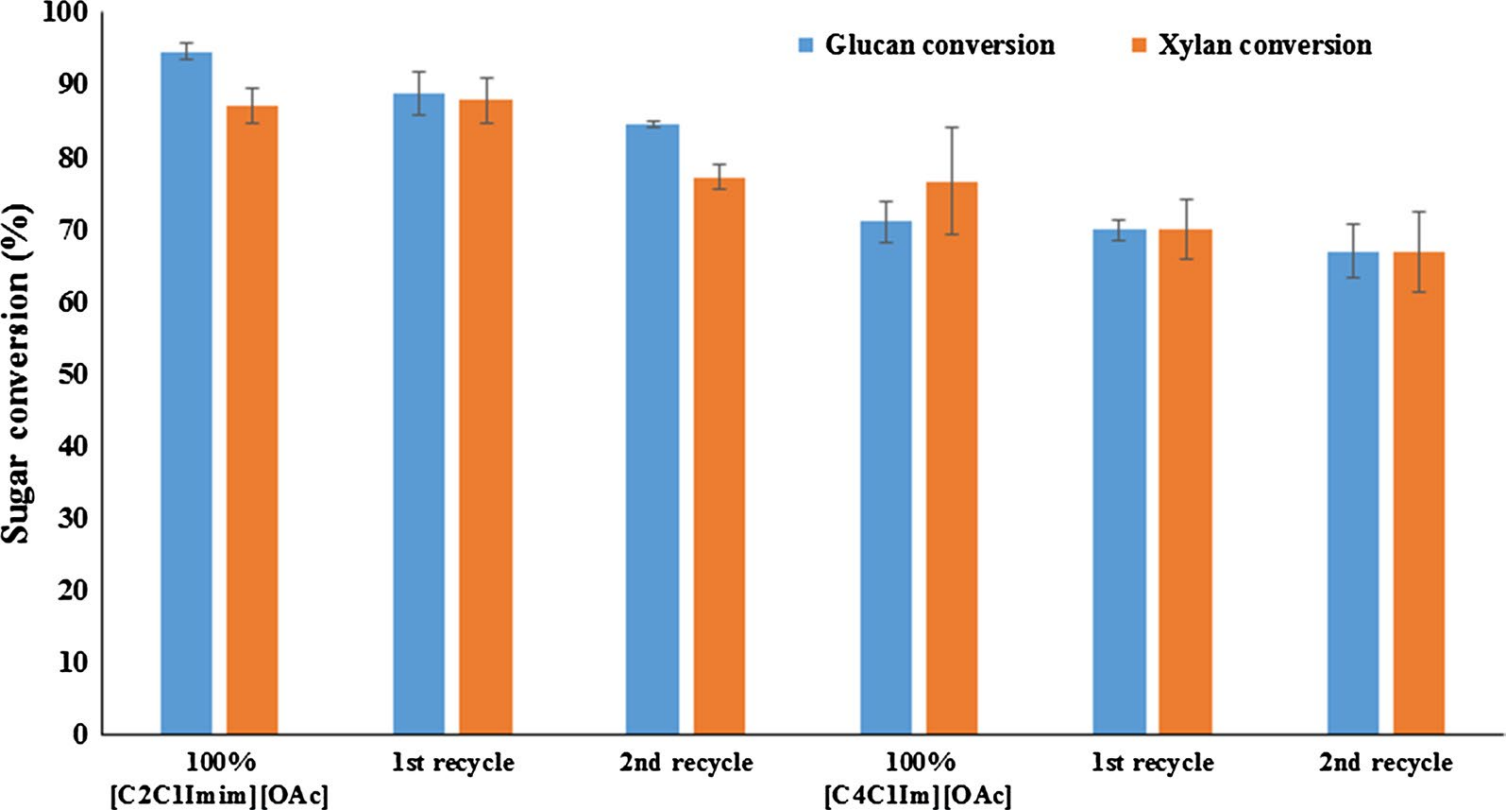
Glucan and xylan conversion of pretreated agave bagasse by recycled [C_2_C_1_Im][OAc] and [C_4_C_1_Im][OAc] on 72-h saccharification time

The CrI obtained from the samples generated by the 100% IL processes is higher than that of those obtained from the recycled samples, although this assessment could be affected by the interference of sharp crystalline peaks of calcium oxalate at 2θ = 15°, 24.5°, and 30.5° [30]. In terms of glucan conversion, [C_2_C_1_Im][OAc] was in the range of ~85 to ~95% in the recycled experiments, while [C_4_C_1_Im][OAc] from ~67 to ~71% (Fig. 7). Similarly, Shill et al. [27] show that a 90% glucan conversion was still maintained using up to 2 recycling steps of [C_2_C_1_Im][OAc] at 140 °C and 1 h using *Miscanthus*. Furthermore, xylan conversion was maintained in a 10% range for both ILs. A significant difference was obtained only on the 2nd recycle of [C_2_C_1_Im][OAc] which did not occur with [C_4_C_1_Im][OAc]. This may be solved with other recycling strategies such as the one recently applied by Sathitsuksanoh et al. [54] that used alcohols as alternative precipitating agents with IL pretreatment process.

## Conclusions

Ternary IL-water systems for the pretreatment of mixed feedstock (such as AGB and MSW) enable delignification and sugar conversion at similar levels to 100% IL. Mixing ILs such as [C_2_C_1_Im][OAc] and [C_4_C_1_Im][OAc] results in an effective method to pretreat biomass with different price ranges while maintaining performance. In addition, effectiveness of [C_2_C_1_Im][OAc] and [C_4_C_1_Im][OAc] during biomass pretreatment remains intact with up to 40% water content. MSW presents relatively higher sugar yield than AGB, whereas the AGB/MSW (1:1) blend shows a glucan conversion of 94.1 and 83.0% using an IL system with ~10 and ~40% water content, respectively. Dissolution of biomass cellulose was also efficient using recycled ILs with only ~10% decrease in glucan, and xylan conversion yields were observed when a 2nd IL recycle was used in comparison with fresh IL. The same effect occurred with cellulose crystallinity of IL-treated biomass where comparable results were obtained when pure and recycle ILs were employed. The chemical structures of neat and recycled ILs demonstrate strong similarities in their behavior, as observed by FTIR and ^1^H-NMR spectroscopy. Altogether, this study highlights the potential of blending MSW as a potentially low-cost feedstock, as using IL-water systems with imidazolium-based ILs mixtures yield comparable biomass treatment results as with pure ILs. Finally, the promising IL recycling results indicate that this strategy can be used and further integrated with downstream saccharification and fermentation within a biorefinery scheme to reduce total operation costs.

## Additional files

**Additional file 1.** FTIR spectra of untreated and pretreated biomass under different ionic liquid–water systems. Unt: untreated, AGB: agave bagasse, MSW: municipal solid waste, Blend: agave bagasse/municipal solid waste (1:1) blend. FTIR spectra of all untreated and pretreated samples from agave bagasse, municipal solid waste and the agave bagasse/municipal solid waste (1:1) blend between 800 and 2000 cm^−1^ with a spectral resolution of 4 cm^−1^.

**Additional file 2.** Chemical changes tracked of fresh and recycled [C_2_C_1_Im][OAc] (up) and [C_4_C_1_Im][OAc] (down). FTIR spectra of recycled ionic liquids [C_2_C_1_Im][OAc] and [C_4_C_1_Im][OAc] from three different cycles.

**Additional file 3.** XRD spectrum and crystallinity index (CrI) of agave bagasse under different conditions (untreated, Fresh IL pretreated and IL-recycled). XRD diffractograms of untreated and pretreated agave bagasse under different process conditions.

## Abbreviations

[C_2_C_1_Im][OAc]: 1-ethyl-3-methylimidazolium acetate
[C_4_C_1_Im][OAc]: 1-butyl-3-methylimidazolium acetate
AGB: agave bagasse
DI: deionized
FTIR: Fourier Transform Infrared spectroscopy
HPLC: high-performance liquid chromatography
IL: ionic liquid
MSW: municipal solid waste
NMR: nuclear magnetic resonance
