# Supplementary figures and images for: Ternary ionic liquid–water pretreatment systems of an agave bagasse and municipal solid waste blend
Source: Biotechnol Biofuels. 2017 Mar 21;10:72. doi: 10.1186/s13068-017-0758-4 (PMC5361851; doi:10.1186/s13068-017-0758-4)

ADDITIONAL FILE 1

| 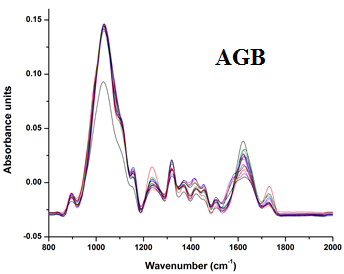 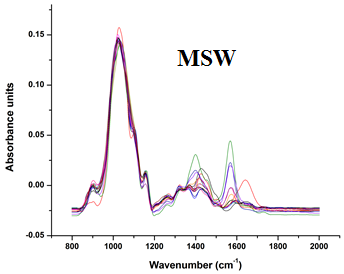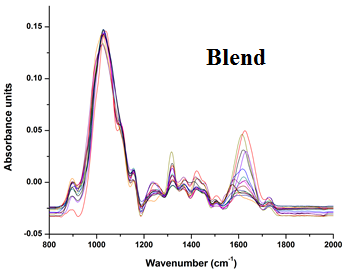 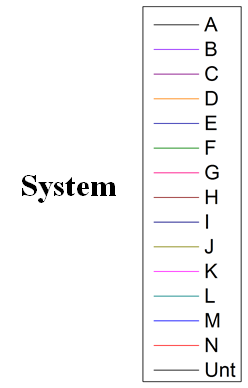 |
| --- |
|  |

Supplement: Supplementary file 1 — Additional file 1. FTIR spectra of untreated and pretreated biomass under different ionic liquid–water systems. Unt: untreated, AGB: agave bagasse, MSW: municipal solid waste, Blend: agave bagasse/municipal solid waste (1:1) blend. FTIR spectra of all untreated and pretreated samples from agave bagasse, municipal solid waste and the agave bagasse/municipal solid waste (1:1) blend between 800 and 2000 cm−1 with a spectral resolution of 4 cm−1. [file 13068_2017_758_MOESM1_ESM.docx]

**ADDITIONAL FILE 2**

| 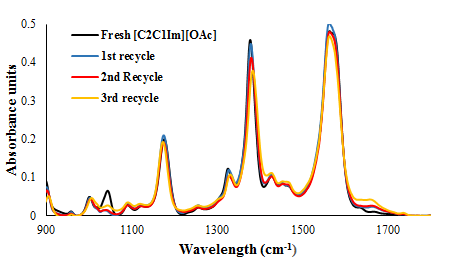 |
| --- |
| 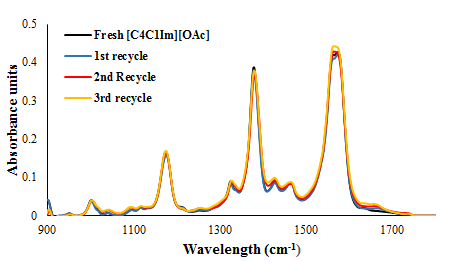 |
|  |

Supplement: Supplementary file 2 — Additional file 2. Chemical changes tracked of fresh and recycled [C2C1Im][OAc] (up) and [C4C1Im][OAc] (down). FTIR spectra of recycled ionic liquids [C2C1Im][OAc] and [C4C1Im][OAc] from three different cycles. [file 13068_2017_758_MOESM2_ESM.docx]

**ADDITIONAL FILE 3**

| 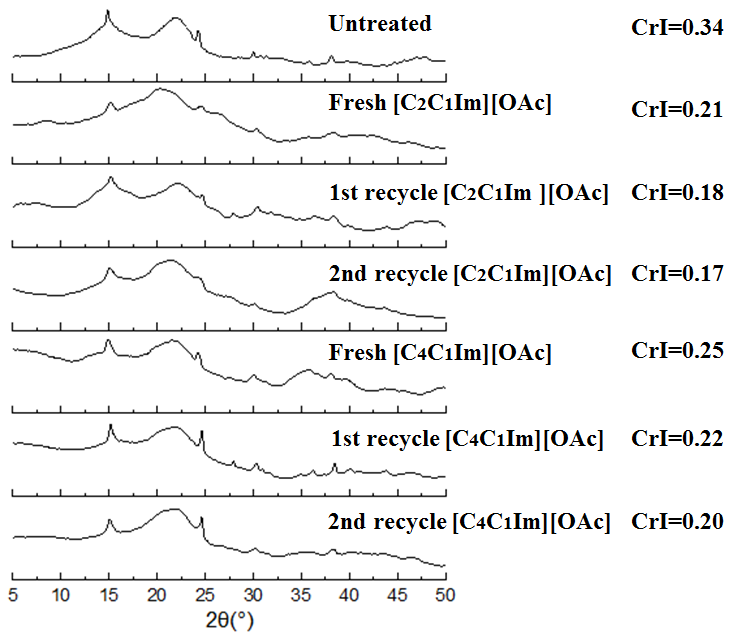 |
| --- |
|  |

Supplement: Supplementary file 3 — Additional file 3. XRD spectrum and crystallinity index (CrI) of agave bagasse under different conditions (untreated, Fresh IL pretreated and IL-recycled). XRD diffractograms of untreated and pretreated agave bagasse under different process conditions. [file 13068_2017_758_MOESM3_ESM.docx]
